# Validation of LILR antibody specificities and development of LILRA3-specific antibodies

**DOI:** 10.3389/fimmu.2026.1729905

**Published:** 2026-04-30

**Authors:** Hiromu Tanimoto, Thi Thu Thao Nguyen, Gen Hasegawa, Yuko Hashikawa, Rikinari Hanayama, Kouyuki Hirayasu

**Affiliations:** 1 Department of Evolutionary Immunology, Environmental Stress Research Center, Kanazawa University, Kanazawa, Ishikawa, Japan; 2 Department of Immunology, Graduate School of Medical Sciences, Kanazawa University, Kanazawa, Ishikawa, Japan; 3 Department of Evolutionary Immunology, Graduate School of Advanced Preventive Medical Sciences, Kanazawa University, Kanazawa, Ishikawa, Japan; 4 WPI Nano Life Science Institute (NanoLSI), Kanazawa University, Kanazawa, Ishikawa, Japan; 5 Advanced Preventive Medical Sciences Research Center, Kanazawa University, Kanazawa, Ishikawa, Japan

**Keywords:** Antibody specificity, cross-reactivity, ELISA, LILR, LILRA3

## Abstract

Leukocyte immunoglobulin (Ig)-like receptors (LILRs) constitute a family of 11 structurally related receptors predominantly expressed on immune cells. Despite their functional diversity, LILRs show a high degree of sequence homology within their extracellular domains, with amino acid sequence identities ranging from approximately 50% to nearly 100%. This molecular similarity poses a significant challenge for antibody-based detection, often resulting in cross-reactivity and misinterpretation of expression profiles. Nevertheless, a comprehensive assessment of antibody specificity across the entire LILR family has not been previously conducted. In this study, we performed a rigorous validation of commercially available antibodies to evaluate their specificity and cross-reactivity against all LILR family members. Using flow cytometry with LILR-transfected K562 cells, we reliably identified specific antibodies for LILRA2, LILRA4, LILRA5, LILRB1, LILRB2, and LILRB4. Notably, more than half of the commercial antibodies showed cross-reactivity. Given the lack of a reliable antibody for LILRA3, the only known soluble member of the family, we generated novel anti-LILRA3 monoclonal antibodies in mice. Three hybridoma clones exhibiting high specificity for LILRA3 were successfully isolated and validated. Using these antibodies, we established a sensitive sandwich ELISA, which successfully detected LILRA3 in individuals carrying functional alleles, while no protein was detected in those with the 6.7-kb deletion or premature termination codons. Moreover, serum concentrations of LILRA3 were substantially higher than previously reported. These findings not only provide essential tools for accurate detection of LILRA3 but also underscore the importance of rigorous antibody validation in LILR-related research.

## 1 Introduction

Leukocyte immunoglobulin (Ig)-like receptors (LILRs) comprise a family of 11 different receptors primarily expressed on immune cells, particularly within the myeloid lineage, although certain members are also detected on lymphocytes, and non-immune cells (1). The LILR family is subdivided into activating receptors (LILRA1, LILRA2, LILRA4, LILRA5, and LILRA6) and inhibitory receptors (LILRB1 through LILRB5). LILRA3 is unique, functioning as a secreted molecule without a transmembrane domain. Although LILRAs lack intrinsic signaling motifs, they associate with FcRγ, an adaptor protein containing immunoreceptor tyrosine-based activation motifs (ITAMs). Conversely, LILRBs possess immunoreceptor tyrosine-based inhibitory motifs (ITIMs) within their intracellular domains.

LILRs can be further classified into two structural groups based on their similarity to LILRB1, which recognizes HLA class I as a ligand (2). Group 1 LILRs (LILRA1, LILRA2, LILRA3, LILRB1, and LILRB2) are characterized by amino acid sequences suggestive of HLA class I recognition, although LILRA1, LILRA2, and LILRA3 exhibit limited or no interaction with HLA class I molecules (3, 4). In contrast, Group 2 LILRs (LILRA4, LILRA5, LILRA6, LILRB3, LILRB4, and LILRB5) do not exhibit the structural features necessary for HLA class I binding.

Each member of the LILR family displays a high degree of homology in their extracellular domains, with amino acid sequence identities ranging from approximately 50% to nearly 100%. Despite this structural similarity, individual LILRs exhibit specific ligand recognition capabilities. For example, while both LILRB1 and LILRB2 recognize HLA class I molecules, LILRB1 does not interact with HLA molecules that lack β2-microglobulin (5). Furthermore, *Plasmodium falciparum* RIFINs, which bear no structural resemblance to HLA class I molecules, have evolved to bind LILRB1 as an immune evasion strategy (6). In another example, LILRA2 uniquely recognizes microbially cleaved immunoglobulins as ligands, despite being classified within Group 1 LILRs (7). These observations highlight the intricate balance between specificity and cross-reactivity that characterizes the LILR family, underscoring the complexity of their immunological functions.

Accumulating evidence has implicated LILRs in a wide variety of pathological processes, prompting growing interest in their immunological roles and regulatory mechanisms (8). However, discrepancies in reported findings have been observed across studies. For instance, studies investigating LILRB5 expression have yielded conflicting results, with some identifying CD14-positive monocytes as the predominant source (9) while others report expression primarily in CD4- and CD8-positive T cells (10). Such discrepancies may be attributable to the use of non-specific antibodies and resultant cross-reactivity, as antibody clones vary across studies and are often insufficiently validated. This challenge is largely attributable to the substantial sequence homology within the extracellular Ig-like domains of LILR family members, which complicates the development of highly specific antibodies targeting individual receptors. Although certain studies have validated antibody cross-reactivity, a comprehensive and systematic evaluation of antibody specificity across the entire LILR family is currently lacking. Therefore, the present study aims to rigorously assess the binding specificity of commercially available LILR antibodies against all known members of the LILR family. Additionally, antibody clones for the LILR family members that lacked the specific antibody were developed in this study.

## 2 Materials and methods

### 2.1 Ethics

This study was reviewed and approved by the Research Ethics Committee of Kanazawa University. Written informed consent was obtained from all participants prior to sample collection. The animal study was reviewed and approved by Institutional Animal Care and Use Committee of Kanazawa University.

### 2.2 Human samples

Genomic DNA samples were obtained from volunteers affiliated with Kanazawa University. Saliva samples were collected using the Oragene-DISCOVER DNA collection kit (DNA Genotek Inc.), from which genomic DNA was extracted according to the manufacturer’s instructions. Serum samples were collected using BD Vacutainer SST II Advance Tubes. Genotyping of LILRA3 alleles, including the functional allele, the 6.7-kb deletion, and non-functional alleles containing premature termination codons, was performed as previously described (11).

### 2.3 Cells

K562 cells, which lack endogenous expression of both LILRs and HLA class I, the well-characterized ligand for several LILRs, were selected for transfection with *LILR* genes to facilitate the evaluation of antibody specificity. LILR-expressing K562 cells were generated using the pMxs-Puro retroviral expression system as previously described (7). Full-length LILR constructs were introduced into K562 cells, and for LILRAs, human FcRγ was co-transfected to enable surface expression. As LILRA3 is a secreted molecule lacking a transmembrane domain, a modified construct was engineered by fusing the LILRB1 transmembrane domain lacking intracellular signaling motifs to the C-terminus of LILRA3, thereby enabling its stable surface expression on K562 cells. The fused amino acid sequence is as follows: RHLGVVIGILVAVILLLLLLLLLFLILRHRRQGKHWTSTQRK. For domain mapping of the newly generated anti-LILRA3 monoclonal antibodies, truncated LILRA3 constructs encoding individual domain combinations, LILRA3-D1D2, D2D3, and D3D4, were generated and expressed in 293T cells via PEI-mediated co-transfection with GFP. Domain definitions were based on a previous report (12).

### 2.4 Flow cytometry

LILR-expressing K562 cells were seeded into a 96-well plate at a density of 1 × 10^5^ cells per well, followed by the addition of respective antibodies. For unlabeled antibodies, APC-conjugated secondary antibodies were added after washing with PBS containing 0.1% bovine serum albumin (BSA, Fujifilm). Subsequent washes were performed using the same washing buffer, after which a propidium iodide (PI, Dojindo) solution at a concentration of 0.4 µg/mL was added to the wells. Flow cytometric analysis was conducted using the MACSQuant Analyzer 10 (Miltenyi Biotec), and data were analyzed using FlowJo software (BD Biosciences). The commercially available antibodies employed in this study, along with their respective concentrations, are listed in **Supplementary Table 1**.

### 2.5 Generation of anti-LILRA3 monoclonal antibodies

Recombinant mouse IgG-Fc–fused LILRA3 protein was produced in 293T cells and purified using rProtein A Sepharose Fast Flow (Cytiva). Female BALB/c mice were immunized three times with the purified fusion protein emulsified in TiterMax Gold adjuvant (TiterMax). Following the final immunization, spleens and draining lymph nodes were harvested, and CD138^+^ plasma cells were enriched using EasySep Mouse CD138 Positive Selection Kit (STEMCELL Technologies). Enriched plasma cells were fused with SP2/0 myeloma cells using polyethylene glycol to generate hybridomas. Hybridoma clones were selected by hypoxanthine-aminopterin-thymidine (HAT) selection and subsequently subjected to limiting dilution to isolate monoclonal populations. Screening for LILRA3-specific antibody production was performed by flow cytometry using LILRA3-expressing K562 cells. Clones exhibiting specific binding to cell-surface LILRA3 were selected for further characterization. Mouse antibody isotypes were determined using the Rapid Monoclonal Antibody Isotyping Kit-2 (Antagen Pharmaceuticals).

### 2.6 Enzyme-linked immunosorbent assay

To evaluate the specificity and potential cross-reactivity of the anti-LILRA3 antibody clone 2E9, which is commonly used for detecting the soluble form of LILRA3 in human serum (13), a direct ELISA was conducted. Briefly, recombinant LILR-Fc fusion proteins (7) were immobilized on high-binding 96-well ELISA plate (IWAKI). Following washing with PBS-T (0.05% Tween 20 in PBS), the anti-LILRA3 antibody clone 2E9 was added and incubated for 1 hour at room temperature. Subsequent to additional washes with PBS-T, the wells were blocked with assay diluent (BioLegend) for 1 hour at room temperature. After a final wash, Peroxidase AffiniPure Donkey Anti-Mouse IgG (H+L) (Jackson) was added, followed by POD Substrate TMB Kit (Nacalai). Absorbance was measured at 450 nm using an Enspire plate reader (PerkinElmer).

To quantitatively assess serum concentrations of soluble LILRA3, a sandwich ELISA was established using monoclonal antibodies generated in this study. High-binding 96-well ELISA plates (IWAKI) were coated overnight at 4 °C with purified anti-LILRA3 monoclonal antibody (clone 4-9B2-4A, 10 µg/mL) diluted in PBS. After blocking with assay diluent (BioLegend) for 1 hour at room temperature, serially diluted recombinant LILRA3 standards and serum samples diluted 1:100 in assay diluent were added and incubated for 1 hour at room temperature. Plates were then extensively washed with PBS-T, followed by incubation with biotinylated anti-LILRA3 monoclonal antibody (clone 2-4H1-6B, 0.5 µg/mL) for 1 hour. After washing, High Sensitivity Streptavidin-HRP (Thermo Scientific) was added and incubated for 30 minutes. Signal development was performed using the TMB Substrate Set (BioLegend), and the reaction was terminated with 2N sulfuric acid (Wako). Absorbance was measured at 450 nm using an Enspire plate reader (PerkinElmer). A standard curve was generated using serial dilutions of recombinant LILRA3, and sample concentrations were calculated using GraphPad Prism software. Biotinylation of the anti-LILRA3 monoclonal antibody (clone 2-4H1-6B) was performed using the Biotin Labeling Kit (Dojindo), following the manufacturer’s instructions. Recombinant LILRA3 used as the standard, which contains a C-terminal DYKDDDDK-tag, was produced in 293T cells and purified using Anti-DYKDDDDK Tag Antibody Beads (Fujifilm), in accordance with the manufacturer’s protocol.

## 3 Results

### 3.1 Validation of anti-LILR antibody specificity

**Figure 1:**
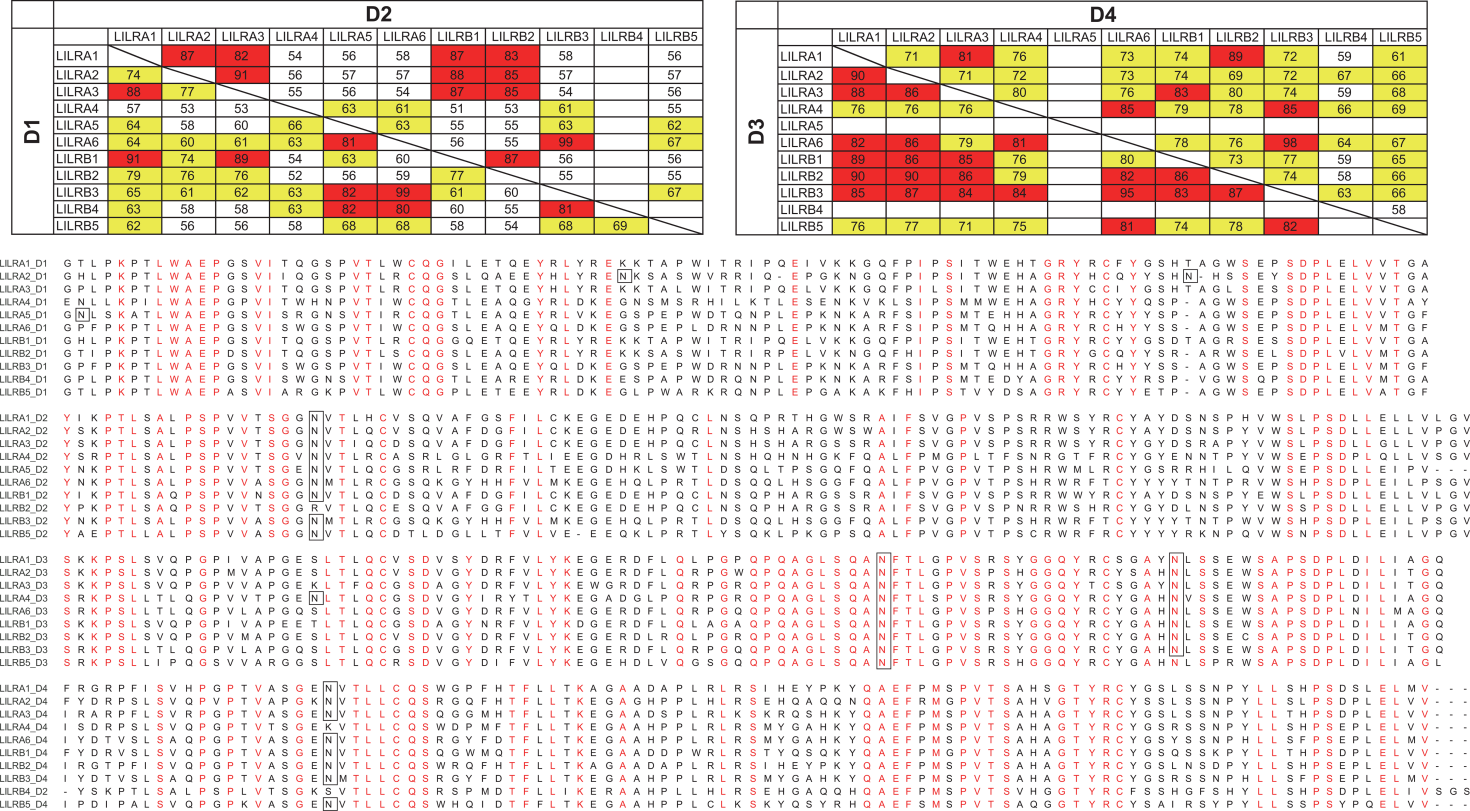
Amino acid sequence homology among Ig-like domains of the LILR family. Top: Pairwise amino acid sequence identities between Ig-like domains of LILR family members are shown in each box. For LILRB4, the membrane-proximal D2 domain was compared with the D4 domains of other family members due to their evolutionary correspondence (22). The D1 and D2 domains of LILRA5 were compared with the corresponding domains of other LILRs. Percent identities of <60%, 60–80%, and >80% are highlighted in white, yellow, and red, respectively. Sequence identity calculations were performed using Clustal Omega. Bottom: Multiple sequence alignment of the extracellular domains (D1–D4) of all LILR family members. Conserved residues are highlighted in red, and predicted N−glycosylation motifs (N−X−S/T) are indicated with boxes in the alignment. N−glycosylation motifs (N−X−S/T) were predicted using NetNGlyc 1.0, and sites with a prediction score above 0.5 were considered positive.

**Figure 2:**
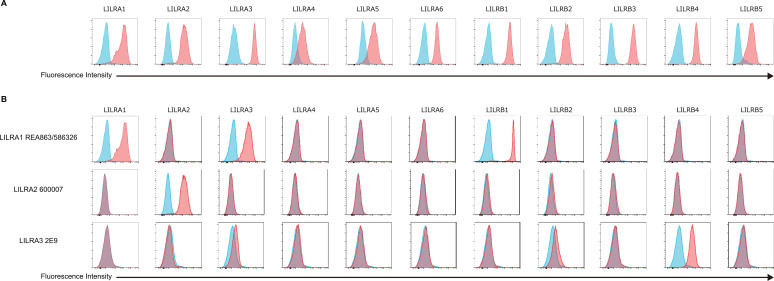
Flow cytometric analysis of antibody reactivity against LILR-expressing K562 cells. **(A)** Expression of individual LILRs on transfected K562 cells was confirmed by flow cytometry using the following antibodies: LILRB1, clone GHI/75; LILRB2, clone 42D1; LILRB3, clone MKT5.1; LILRB4, clone ZM4.1; LILRB5, clone 395239; LILRA1, clone 586326; LILRA2, clone 600007; LILRA3, rabbit polyclonal antibody; LILRA4, clone 17G10.2; LILRA5, clone 711828; LILRA6, clone 921330. **(B)** Representative examples of cross-reactivity (anti-LILRA1 antibody clone 586326), specific reactivity (anti-LILRA2 antibody clone 600007), and weak reactivity (anti-LILRA3 antibody clone 2E9). Blue histograms represent parental K562 cells; red histograms represent LILR-expressing cells.

**Figure 3:**
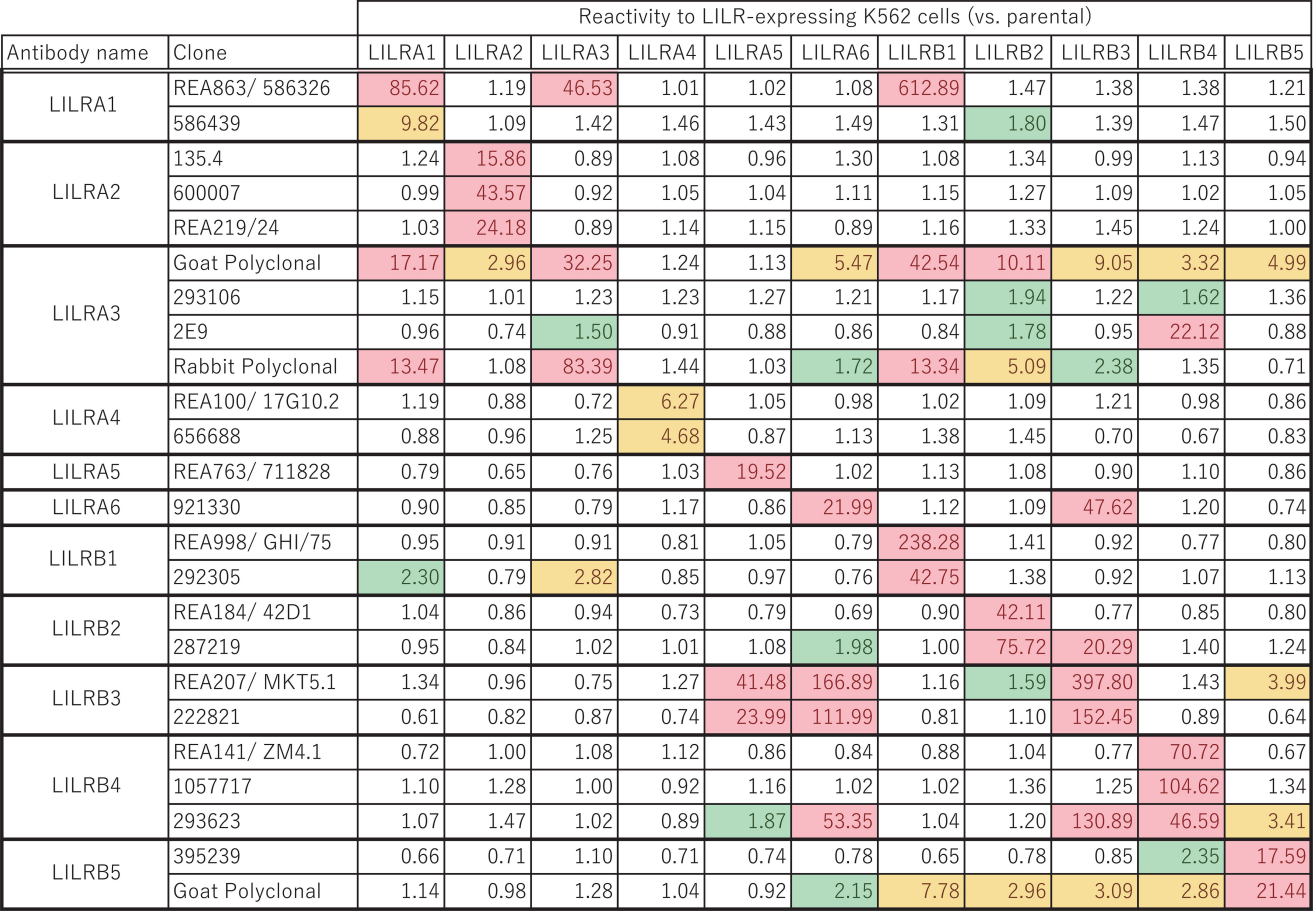
Comprehensive validation of anti-LILR antibody specificity. Mean fluorescence intensity (MFI) values for each antibody across LILR-expressing K562 cell lines, relative to parent K562 cells, are shown. Fold changes of 1.5–2.5, 2.5–10, and >10 are highlighted in green, yellow, and red, respectively.

**Figure 4:**
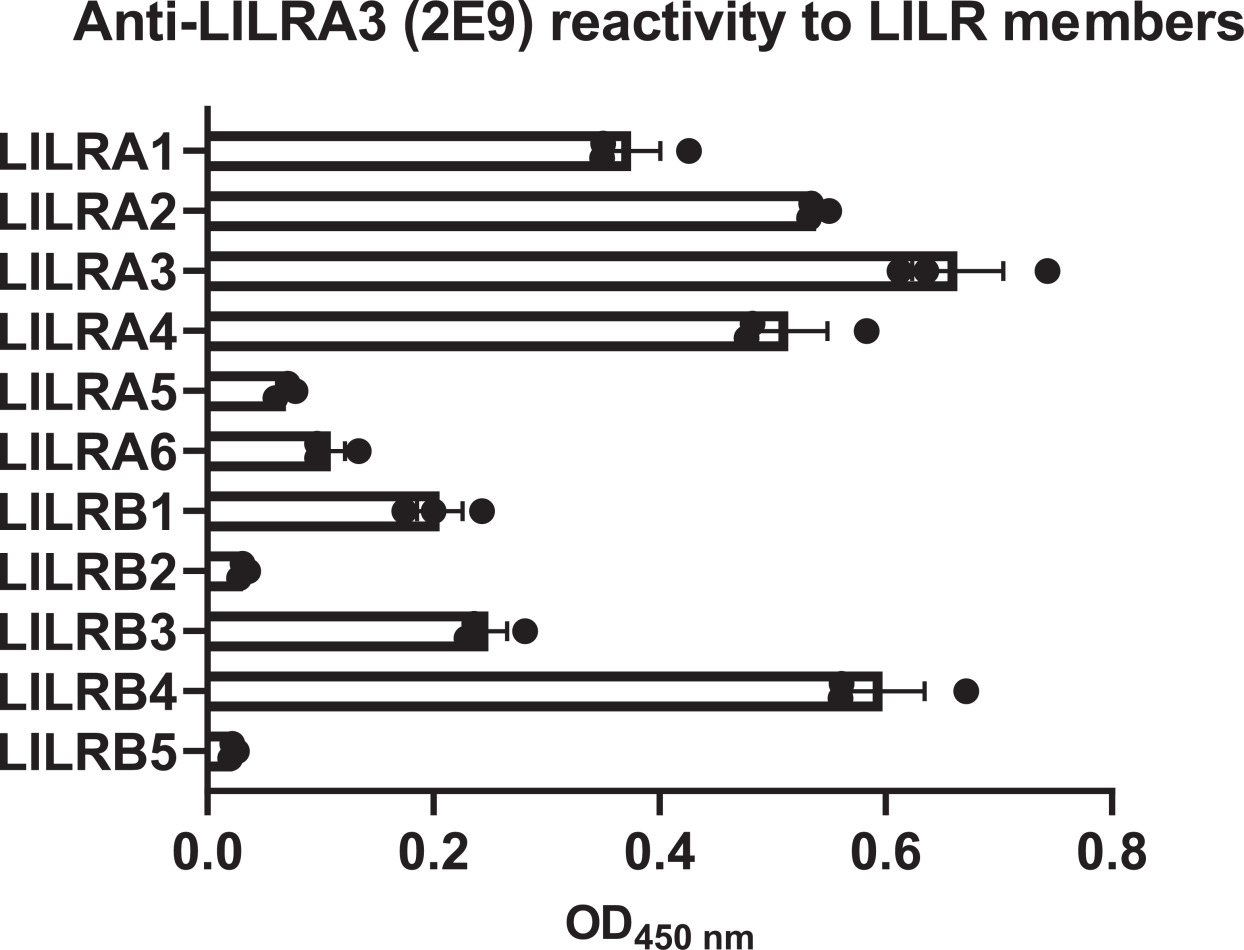
Direct ELISA assessing cross-reactivity of anti-LILRA3 antibody clone 2E9. Recombinant proteins representing each LILR family member were immobilized on a 96-well ELISA plate. A direct ELISA was performed using anti-LILRA3 antibody clone 2E9. The y-axis indicates the LILR recombinant proteins, and the x-axis shows absorbance at 450 nm, reflecting antibody binding. Data represent mean ± SEM of triplicate measurements, representative of at least two independent experiments. Statistical significance was determined by one−way ANOVA (*P*< 0.001).

The high degree of homology observed among the Ig-like domains of LILRs (**Figure 1**) suggests the potential for cross-reactivity in antibodies targeting individual LILR molecules. To rigorously evaluate antibody specificity for each LILR, we established cell lines individually expressing each LILR. The K562 cell line, which lacks endogenous expression of both LILRs and HLA class I, the well-characterized ligand for several LILRs, was selected for this purpose. Given that LILRA3 is a soluble form lacking a transmembrane domain, the LILRB1 transmembrane domain was fused to the C-terminus of LILRA3, thereby enabling its stable expression on the cell surface. Flow cytometric analysis confirmed that each LILR was independently and sufficiently expressed in these cells (**Figure 2A**). Subsequently, a total of 24 commercially available anti-LILR antibodies were evaluated for their reactivity against LILR-expressing K562 cells using flow cytometry (**Supplementary Table 1**). As shown in **Figures 2B**, **3**, while several antibodies exhibited specificity toward individual LILRs, others demonstrated cross-reactivity with multiple family members (**Supplementary Figure 1**). To quantitatively assess cross-reactivity, mean fluorescence intensity (MFI) values were compared across the LILR-expressing cell lines. To assist interpretation, fold−change values were grouped into three ranges (1.5–2.5, 2.5–10, and >10). Because flow−cytometry MFI values can vary between experimental days due to technical and cellular factors, fold−change values within the 1.5–2.5 range showed variability across independent experiments (**Figure 3**; **Supplementary Figure 2**). Therefore, only antibodies that reproducibly exhibited ≥2.5−fold increases exclusively against the target LILR in two independent experiments were considered highly reliable. Based on this criterion, 8 out of the 24 antibodies showed high specificity (**Figure 3**; **Supplementary Figures 1**, **2**). Namely, highly specific antibodies were identified for LILRA2 (clone 135.4, and 600007), LILRA4 (clone 17G10.2), LILRA5 (clone 711828), LILRB1 (clone GHI/75), LILRB2 (clone 42D1), and LILRB4 (clone ZM4.1, and 1057717). It should be noted that the LILRA3-2E9 antibody, which is commonly recommended for ELISA-based detection of soluble LILRA3, exhibited weak reactivity to LILRA3 and strong cross-reactivity with LILRB4 (**Figure 2B**). To further investigate this cross-reactivity, a direct ELISA was performed. As shown in **Figure 4**, the LILRA3-2E9 antibody demonstrated cross-reactivity with multiple LILRs, including LILRA2, LILRA4, and LILRB4. Although several N−glycosylation motifs have been previously reported in LILRA3 (14), no clear pattern was observed that could explain the broad antibody cross−reactivity (**Figure 1**). However, because the presence and structural impact of glycans may vary among receptors, we cannot fully exclude the possibility that glycosylation contributes to cross−reactivity. Collectively, these findings reveal the substantial variability in specificity among commercially available anti-LILR antibodies and underscore the importance of thorough validation to ensure accurate interpretation of LILR expression and function.

### 3.2 Development of highly specific anti-LILRA3 monoclonal antibodies

**Figure 5:**
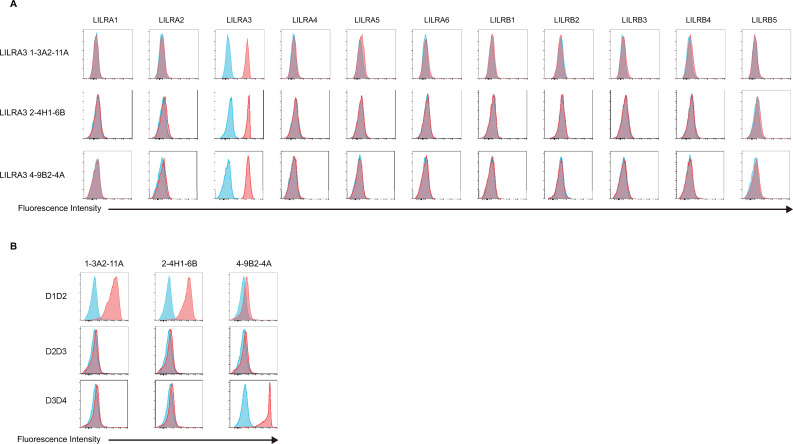
Characterization of newly generated anti-LILRA3 monoclonal antibodies. **(A)** Flow cytometry histograms showing reactivity of three hybridoma clones, 1-3A2-11A (mouse IgG2a, κ), 2-4H1-6B (mouse IgG2b, κ), and 4-9B2-4A (mouse IgG1, κ), against LILR-expressing K562 cells. Blue histograms represent parental K562 cells; red histograms represent LILR-expressing cells. **(B)** Domain mapping of antibody recognition using truncated LILRA3 constructs (D1D2, D2D3, D3D4) expressed in 293T cells via transfection. Blue histograms represent parental 293T cells; red histograms represent truncated LILRA3-expressing cells.

**Figure 6:**
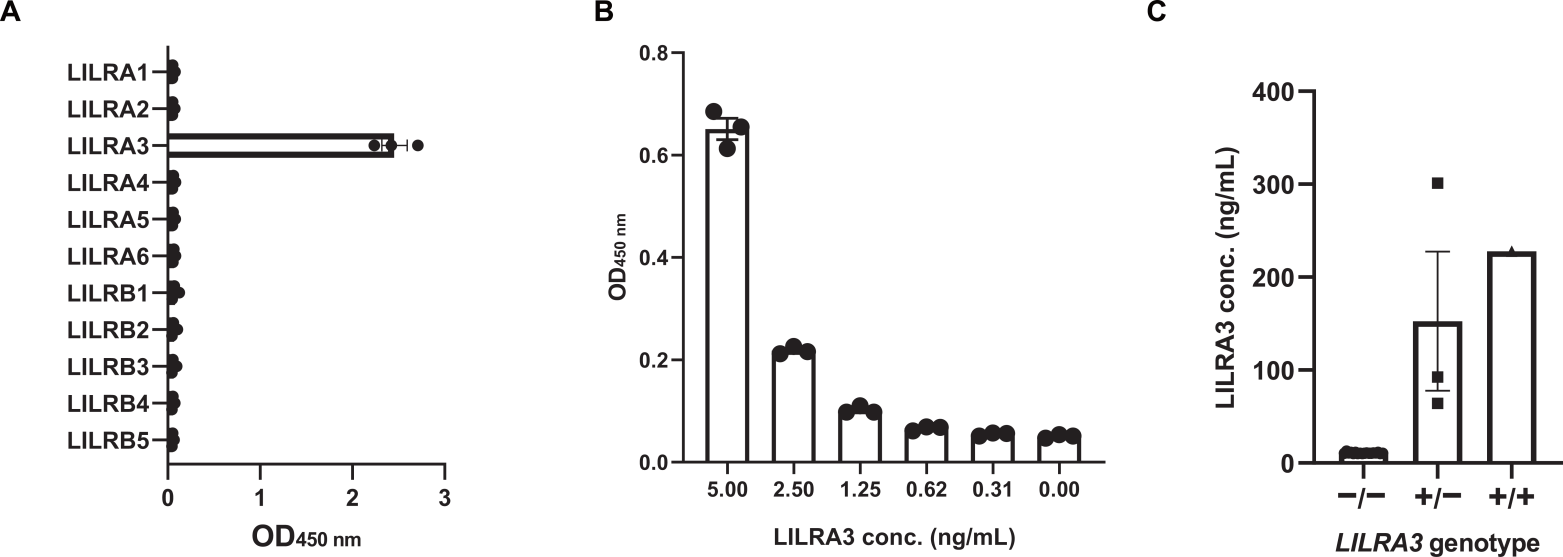
Sensitive and specific detection of soluble LILRA3 using a sandwich ELISA with newly generated monoclonal antibodies. **(A)** Specific detection of LILRA3 using a sandwich ELISA composed of clone 4-9B2-4A as the capture antibody and biotinylated clone 2-4H1-6B as the detection antibody. The y-axis indicates the LILR recombinant proteins (1 μg/mL), and the x-axis shows absorbance at 450 nm, reflecting antibody binding. **(B)** Sensitivity of the established sandwich ELISA was evaluated using serial dilutions of recombinant LILRA3 protein. **(C)** Application of the sandwich ELISA to human serum samples. Genotypes are indicated as follows: –/– (homozygous for non-functional LILRA3 alleles), +/– (heterozygous), and +/+ (homozygous for functional LILRA3 alleles). Serum LILRA3 concentrations were measured and compared across genotypes. Data represent mean ± SEM of duplicate measurements, representative of at least two independent experiments.

Among the LILRs lacking specific antibodies, LILRA3 was prioritized because it is the only soluble member of the LILR family, serves as a potential circulating biomarker, and has documented associations with several diseases. Therefore, we generated novel anti-LILRA3 monoclonal antibodies in mice. Four hybridoma clones were successfully isolated: 1-3A2-11A (mouse IgG2a, κ), 2-4H1-6B (mouse IgG2b, κ), 4-9B2-4A (mouse IgG1, κ), and 5-9E2-1G (mouse IgG1, κ) (**Table 1**). Among these, clone 5-9E2-1G exhibited cross-reactivity with LILRA6 and LILRB3 (**Supplementary Figure 3**), and was therefore excluded from further analysis. The remaining three clones demonstrated high specificity for LILRA3 (**Figure 5A**) and were selected for subsequent characterization. To determine the recognition domain of these antibodies, domain mapping was performed using 293T cells individually expressing truncated forms of LILRA3-D1D2, -D2D3, and -D3D4. Clones 1-3A2-11A and 2-4H1-6B selectively recognized cells expressing the LILRA3-D1D2, but not D2D3 or D3D4, indicating that these antibodies target domain 1 of LILRA3 (**Figure 5B**). In contrast, clone 4-9B2-4A recognized the LILRA3-D3D4, but not D1D2 or D2D3, indicating its specificity for domain 4 (**Figure 5B**). Based on these domain-specific binding profiles, we established a highly sensitive sandwich ELISA using a combination of D1- and D4-targeting antibodies. As shown in **Supplementary Figure 4**, the combination of clone 4-9B2-4A as the capture antibody and biotinylated clone 2-4H1-6B as the detection antibody efficiently detected recombinant LILRA3. Therefore, this antibody pair was selected for subsequent analyzes. Upon optimization, the sandwich ELISA specifically detected LILRA3 without cross-reactivity to other LILR family members, demonstrating its utility for selective quantification (**Figure 6A**). The established sandwich ELISA exhibited a lower limit of detection of approximately 620 pg/mL (**Figure 6B**). One notable feature of LILRA3 is the presence of non-functional alleles in the human population, including a 6.7 kb genomic deletion and premature termination codons (11, 15). To evaluate the clinical applicability of the developed sandwich ELISA, we tested its ability to detect LILRA3 in human serum samples. The assay successfully detected LILRA3 in individuals carrying functional alleles, but not in those with non-functional alleles, confirming its specificity in serum (**Figure 6C**; **Supplementary Figure 5**). Notably, serum concentrations of LILRA3 measured using the newly developed sandwich ELISA ranged from approximately 100 to 300 ng/mL, which is substantially higher than previously reported values, typically estimated at 5 to 10 ng/mL (13, 16). This discrepancy suggests that earlier studies may have underestimated LILRA3 levels due to limitations in antibody specificity or assay sensitivity. Collectively, these findings demonstrate the successful generation of highly specific monoclonal antibodies against LILRA3 and the establishment of a reliable and sensitive ELISA platform. This assay provides a valuable tool for investigating the physiological and pathological roles of LILRA3 in humans.

**Table:** Table 1 Summary of the newly generated anti-LILRA3 monoclonal antibodies.

Clone name	Isotype	Specificity	Cross-reactivity	Recognition domain
1-3A2-11A	mouse IgG2a, κ	High	None	D1
2-4H1-6B	mouse IgG2b, κ	High	None	D1
4-9B2-4A	mouse IgG1, κ	High	None	D4
5-9E2-1G	mouse IgG1, κ	Low	LILRA6, LILRB3	D4

## 4 Discussion

This study provides a comprehensive catalog of antibody specificities and cross-reactivities within the LILR family. Our findings revealed that a substantial proportion of commercially available anti-LILR antibodies exhibited cross-reactivity with multiple LILR family members. This is likely due to the high degree of homology in the Ig-like domains within the LILR family. Notably, we were unable to identify specific antibodies for LILRA3, LILRA6, LILRB3, and LILRB5. The lack of specific antibodies for these LILRs may hinder research on their expression patterns and functional roles in immune responses. In contrast, the antibodies against LILRA2, LILRA4, and LILRA5 evaluated in this study displayed selective reactivity, suggesting that these receptors possess distinct structural features relative to other family members.

The absence of specificity observed for anti-LILRA3 antibodies may be explained by its classification within Group 1 LILRs, which share substantial structural similarity, thereby contributing to cross-reactivity among antibodies targeting this subgroup. Likewise, LILRA6 and LILRB3 show the highest homology within the LILR family, precluding discrimination between them. However, a previous study has indicated that LILRA6 and LILRB3 can be distinguished at the allelic level (17), suggesting the feasibility of developing allele-specific antibodies capable of differentiating these closely related receptors. In addition, cross-reactivity was observed between receptors with relatively low sequence homology. For example, anti-LILRA3 monoclonal antibody clone 5-9E2-1G exhibited cross-reactivity with LILRA6 and LILRB3, both classified as Group 2 LILRs, despite being generated against LILRA3, a Group 1 member. This finding indicates that antibody cross-reactivity cannot be accurately predicted based solely on primary sequence similarity. Factors such as conformational epitopes or conserved structural motifs may contribute to unexpected cross-reactivity, even among distantly related receptors. These results underscore the widespread issue of non-specific cross-reactivity among anti-LILR antibodies and emphasize the critical importance of rigorous specificity validation prior to their application in both research and clinical settings. Future efforts will focus on determining the expression sites of these LILRs, potentially through the development of highly specific antibodies.

A notable example of cross-reactivity was observed with the widely used LILRA3-2E9 antibody (13), which demonstrated weak binding to LILRA3 and strong cross-reactivity with LILRB4. This highlights the limitations of currently available reagents and the potential for misinterpretation of LILR expression data in both basic and clinical research. To address this issue, we successfully generated novel anti-LILRA3 monoclonal antibodies and established a highly sensitive sandwich ELISA for the detection of soluble LILRA3 in human serum. Using this assay, we demonstrated that LILRA3 protein is undetectable not only in individuals carrying the 6.7-kb genomic deletion but also in those harboring non-functional alleles with premature termination codons. These results confirm the specificity of the assay and its utility in distinguishing functional from non-functional LILRA3 alleles at the protein level. LILRA3 has been implicated in several pathological conditions, including Takayasu arteritis, prostate cancer, and multiple sclerosis (18–20). Therefore, the ELISA system developed in this study may serve as a valuable tool for investigating the role of LILRA3 in disease pathogenesis, offering insights that may be missed by genetic analyzes alone.

One limitation of this study is the relatively small number of blood samples obtained from healthy donors. This was partly due to the high frequency of non-functional *LILRA3* alleles in the Japanese population (11, 15). Future studies involving a larger and more diverse cohort, including individuals with various diseases and genetic variants, are warranted to identify disease-specific LILR expression patterns and potential associations between *LILR* polymorphisms and disease susceptibility. Another limitation is that antibody specificity was validated primarily by flow cytometry. For example, the reactivity of the anti-LILRA3 antibody clone 2E9 differed between flow cytometry (**Figure 2B**) and ELISA (**Figure 4**). In flow cytometry, clone 2E9 showed weak reactivity to LILRA3-expressing K562 cells, whereas in ELISA, it reacted strongly with recombinant LILRA3. However, its cross-reactivity with LILRB4 was consistently observed in both assays. Therefore, the data presented in this study are specific to flow cytometric analysis, and we do not exclude the possibility that the anti-LILR antibodies used here may be applicable to other assays.

These observations are consistent with a recent report highlighting widespread specificity issues among commercial antibodies (21). Importantly, the cross-reactivity observed in this study should not be interpreted as a criticism of existing reagents, but rather as a reflection of the inherent technical challenges in antibody development. Given the high degree of homology among LILRs, achieving absolute specificity is difficult, and the results presented here emphasize the importance of thorough validation prior to antibody application in experimental settings. Continued collaboration between academic laboratories and antibody manufacturers will be important for improving reagent quality and expanding the repertoire of validated tools. Taken together, this study provides a basis for further investigation into the biological roles of LILRs in human health and disease, and may contribute to the development of more precise diagnostic and therapeutic approaches.

## Data Availability

The original contributions presented in the study are included in the article/**Supplementary Material**. Further inquiries can be directed to the corresponding author.
