# Supplementary material for: Validation of LILR antibody specificities and development of LILRA3-specific antibodies
Source: Front Immunol. 2026 Apr 30;17:1729905. doi: 10.3389/fimmu.2026.1729905 (PMC13171362; doi:10.3389/fimmu.2026.1729905)
Supplement: Supplementary file 1 [file Image1.pdf]

**Supplementary Figure 1**

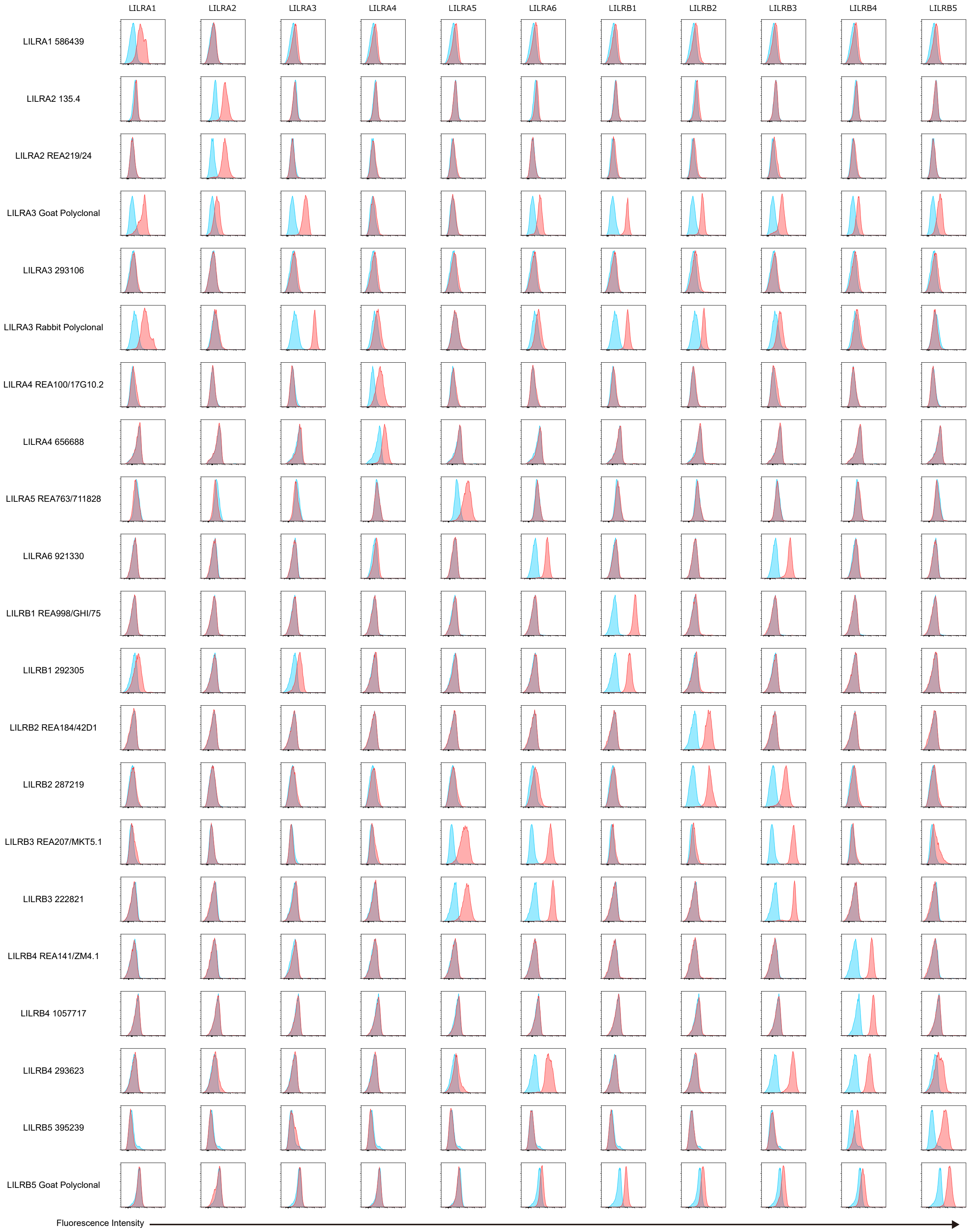


**Supplementary Figure 1. Antibody reactivity histograms for LILR-expressing K562 cells.**
All histogram plots used to calculate the mean fluorescence intensities (MFIs) summarized in Figure 3 are shown.

| Antibody name | Clone | LILRA1 | LILRA2 | LILRA3 | LILRA4 | LILRA5 | LILRA6 | LILRB1 | LILRB2 | LILRB3 | LILRB4 | LILRB5 |
|---|---|---|---|---|---|---|---|---|---|---|---|---|
| LILRA1 | REA863/ 586326 | 316.54 | 0.70 | 86.53 | 1.08 | 0.83 | 0.98 | 942.64 | 0.98 | 0.75 | 0.65 | 0.70 |
| | 586439 | 7.40 | 0.94 | 1.35 | 1.42 | 1.36 | 1.20 | 1.15 | 1.06 | 1.27 | 1.15 | 1.01 |
| LILRA2 | 135.4 | 1.24 | 27.48 | 1.18 | 0.97 | 1.02 | 0.94 | 1.07 | 1.44 | 0.89 | 0.87 | 0.89 |
| | 600007 | 0.62 | 32.39 | 0.69 | 0.92 | 0.71 | 1.31 | 1.49 | 0.69 | 1.14 | 0.84 | 0.92 |
| | REA219/ 24 | 0.91 | 21.41 | 0.82 | 0.93 | 0.84 | 1.32 | 1.72 | 0.85 | 1.44 | 1.54 | 1.41 |
| LILRA3 | Goat Polyclonal | 13.77 | 3.46 | 27.00 | 1.08 | 1.21 | 4.75 | 19.54 | 6.27 | 7.57 | 3.24 | 3.61 |
| | 293106 | 1.12 | 1.41 | 0.85 | 1.11 | 1.13 | 1.11 | 1.04 | 1.25 | 1.24 | 0.97 | 1.03 |
| | 2E9 | 0.82 | 0.60 | 0.96 | 0.72 | 0.79 | 0.66 | 0.70 | 1.00 | 0.74 | 15.72 | 1.73 |
| | Rabbit Polyclonal | 16.27 | 1.35 | 124.52 | 1.69 | 1.57 | 2.37 | 22.93 | 8.07 | 2.74 | 1.64 | 1.00 |
| LILRA4 | REA100/ 17G10.2 | 1.16 | 1.17 | 0.97 | 4.86 | 1.34 | 1.22 | 0.93 | 1.37 | 1.37 | 0.97 | 0.99 |
| | 656688 | 0.59 | 0.54 | 0.47 | 1.70 | 0.53 | 0.61 | 0.63 | 0.89 | 0.78 | 1.03 | 0.96 |
| LILRA5 | REA763/ 711828 | 0.95 | 1.14 | 1.00 | 0.98 | 17.41 | 0.83 | 1.09 | 1.40 | 0.79 | 1.00 | 0.74 |
| LILRA6 | 921330 | 0.98 | 0.89 | 1.03 | 1.92 | 1.12 | 72.05 | 1.14 | 1.40 | 178.11 | 1.29 | 1.07 |
| LILRB1 | REA998/ GHI/75 | 0.36 | 0.36 | 0.37 | 0.47 | 0.42 | 0.48 | 258.69 | 0.41 | 0.39 | 0.43 | 0.38 |
| | 292305 | 3.35 | 0.73 | 54.29 | 1.24 | 1.01 | 1.43 | 395.95 | 1.52 | 1.32 | 1.00 | 0.79 |
| LILRB2 | REA184 42D1 | 0.90 | 0.59 | 0.73 | 1.10 | 0.78 | 1.03 | 1.28 | 75.16 | 0.55 | 0.55 | 0.61 |
| | 287219 | 0.98 | 1.07 | 0.98 | 1.04 | 1.11 | 1.60 | 0.93 | 40.85 | 34.71 | 1.18 | 1.26 |
| LILRB3 | REA207/MKT5.1 | 1.19 | 0.63 | 0.84 | 1.13 | 28.20 | 99.37 | 1.46 | 2.10 | 192.24 | 1.40 | 3.85 |
| | 222821 | 0.62 | 0.61 | 0.65 | 0.62 | 80.92 | 359.49 | 0.66 | 11.54 | 467.10 | 0.96 | 0.63 |
| LILRB4 | REA141/ ZM4.1 | 0.54 | 0.60 | 0.92 | 0.61 | 0.57 | 0.56 | 0.59 | 0.67 | 0.53 | 139.98 | 0.58 |
| | 1057717 | 1.00 | 0.83 | 1.20 | 0.84 | 0.88 | 0.81 | 0.84 | 0.89 | 0.81 | 170.75 | 0.84 |
| | 293623 | 0.72 | 1.19 | 0.73 | 0.59 | 2.74 | 90.20 | 0.67 | 2.16 | 147.89 | 45.80 | 4.63 |
| LILRB5 | 395239 | 0.95 | 1.10 | 1.01 | 0.99 | 1.21 | 1.11 | 1.05 | 1.18 | 1.17 | 2.27 | 6.46 |
| | Goat Polyclonal | 1.65 | 1.53 | 1.53 | 1.17 | 1.32 | 2.65 | 8.23 | 3.59 | 3.40 | 2.72 | 21.26 |

**Supplementary Figure 2. Independent dataset of MFI-based fold-change values for all antibodies across LILR-expressing K562 cell lines**
Mean fluorescence intensity (MFI)–based fold-change values obtained from an independent experiment performed on a separate day are shown. These data provide an additional validation set corresponding to the fold-change results summarized in Figure 3.

## Supplementary Figure 3

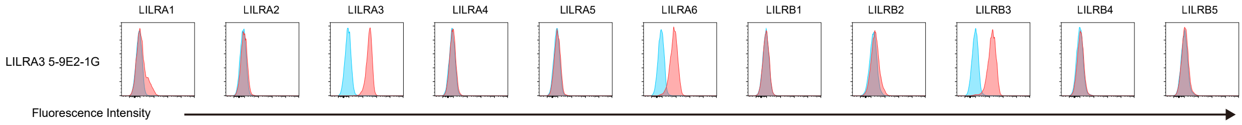


**Supplementary Figure 3. Characterization and cross-reactivity of anti-LILRA3 monoclonal antibody clone 5-9E2-1G**
Flow cytometry histograms showing the reactivity of hybridoma clone 5-9E2-1G (mouse IgG1, κ), against LILR-expressing K562 cells. Blue histograms represent parental K562 cells, while red histograms indicate LILR-expressing K562 cells.

## Supplementary Figure 4

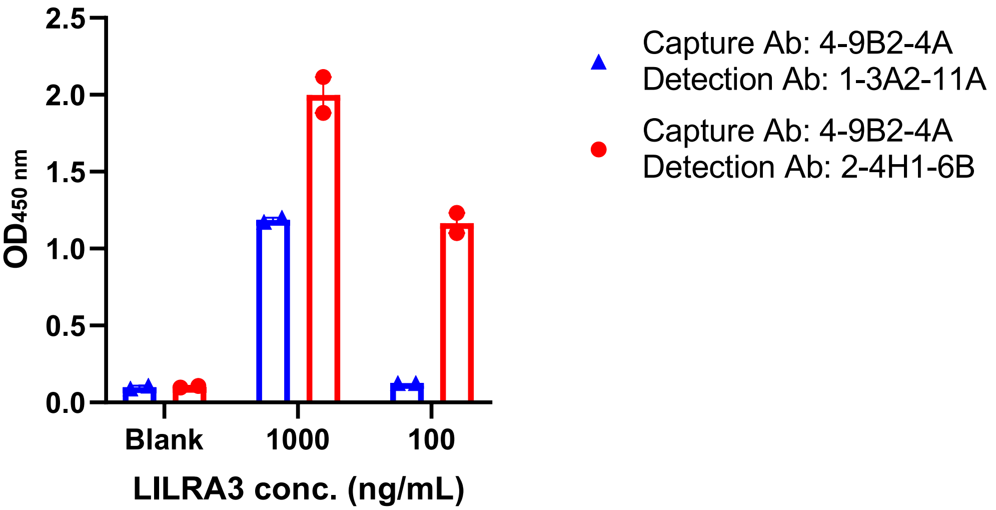


**Supplementary Figure 4. Selection of the antibody pair suitable for sandwich ELISA in newly generated monoclonal antibodies**
To identify the antibody pair with high detection sensitivity, recombinant LILRA3 protein was used as the antigen at concentrations of 1000 ng/mL and 100 ng/mL. The capture antibody (clone 4-9B2-4A) was immobilized on the plate, and biotinylated detection antibodies (clones 1-3A2-11A and 2-4H1-6B) were tested in duplicate. Signal intensities were compared to determine the optimal antibody pair for sandwich ELISA.

## Supplementary Figure 5

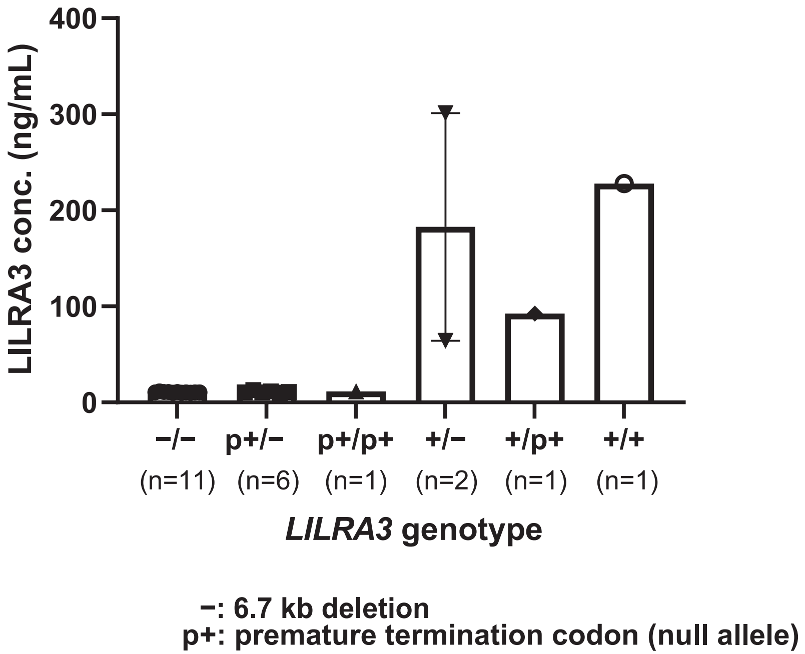


**Supplementary Figure 5. Sensitive and specific detection of soluble LILRA3 by sandwich ELISA across *LILRA3* genotypes**
Application of the sandwich ELISA to human serum samples. Genotypes are indicated as follows: –/– (homozygous for deletion alleles), p+/– (heterozygous for a null allele and a deletion allele), p+/p+ (homozygous for null alleles), +/– (heterozygous for a functional allele and a deletion allele), +/p+ (heterozygous for a functional allele and a null allele), and +/+ (homozygous for functional *LILRA3* alleles). Serum LILRA3 concentrations were measured and compared across genotypes.
